# Dermoscopic Findings in Clinically Diagnosed Cases of Plantar Warts, Corns, and Calluses: A Cross-Sectional Study

**DOI:** 10.7759/cureus.38093

**Published:** 2023-04-25

**Authors:** Sanika Patil, Milind Borkar, Sushil Pande, Kirtee Meshram, Manjiri Oke

**Affiliations:** 1 Department of Dermatology, NKP Salve Institute of Medical Sciences and Research Centre and Lata Mangeshkar Hospital, Nagpur, IND

**Keywords:** palmoplantar, paring, callus, corn, wart, dermoscopy

## Abstract

Introduction:* *With the naked eye, it can frequently be challenging to tell a plantar wart from a corn or callus. A non-invasive diagnostic method called dermoscopy allows for the inspection of morphological features that are not apparent to the unaided eye. This study aimed to examine the dermoscopic findings in pared and unpared cases of palmoplantar warts, corns, and calluses.

Methods: Seventy patients who had palmoplantar warts, corns, and calluses were included in this study. A predesigned structured format was used to document the dermoscopic findings.

Result: The majority of patients (51.4%) had warts followed by callus (28.6%) and corn (20%). On dermoscopic examination, all unpared and pared cases of warts had homogenous black/red dots. Translucent central core was present in 92.85% unpared and 100% pared lesions of corns. Homogenous opacity was present in 75% unpared and 100% pared cases of callus. There was no association between unpared and pared lesions (p>0.05).

Conclusion: The accuracy of identifying various clinical types of cutaneous warts, calluses, and corns can be improved by dermoscopy without paring.

## Introduction

In dermatology practice, papules and plaques that affect the palmar as well as plantar aspect of the hand and foot are frequently seen, which can make it difficult to carry out daily tasks. The most prevalent lesions are calluses, corns, and palmoplantar warts [1]. Warts can occasionally be difficult to distinguish from other skin conditions, such as calluses and corns. Dermoscopy is intended to help distinguish between similar skin lesions, such as palmoplantar warts, corns, and calluses rather than to replace other diagnostic techniques [2]. The distinction between calluses and corns, each of which displays uniform opacity or a translucent center, is also made easier by this technique [3].

In the past few years, dermoscopy has dramatically increased in popularity, and numerous lesions have been investigated. To illuminate a lesion's finer details, the device uses polarized light and magnification. Additionally, polarized light when used in a non-contact fashion can seep through the epidermis with minimal reflection, enabling the visualization of deeper structures. A link between macroscopic clinical dermatology and microscopic dermatopathology is created by dermoscopy which is a non-invasive, straightforward, and reasonably priced diagnostic procedure that allows imaging of morphological aspects that are not visible to the naked eye [4]. Due to paucity of studies on this topic in the literature, we focused on studying the dermoscopic findings in pared and unpared cases of palmoplantar warts, corns, and calluses.

## Materials and methods

After getting written informed consent, 70 patients visiting OPD of tertiary healthcare setting with a clinical diagnosis of palmoplantar warts, corns, or calluses were enrolled in the study. Prior to starting the research, it received approval from the institutional ethics committee. Children less than 12 years of age, pregnant and lactating females, and hypertensive and diabetic patients were excluded from the study. Sample size of 70 was calculated by the following formula: z^2^pq/d^2^, where z=1.96 with 95% confidence interval.

Dermoscopic examinations of the patients were performed both before and after paring. Dermoscopic images were captured using Dino-Lite digital dermatoscope (New Taipei City, Taiwan: AnMo Electronics Corporation) with 10× magnification. All findings were entered in a Microsoft Excel sheet. The significance of variation in the frequency distribution of the data was determined using the chi-square test. Statistical significance was defined as a p-value of 0.05 or lower. Data were expressed as percentage and mean±SD and analyzed using unpaired or paired t-tests as applicable. SPSS version 21.0 for Windows (Armonk, NY: IBM Corp) was used to perform the statistical analysis.

## Results

**Figure 1:**
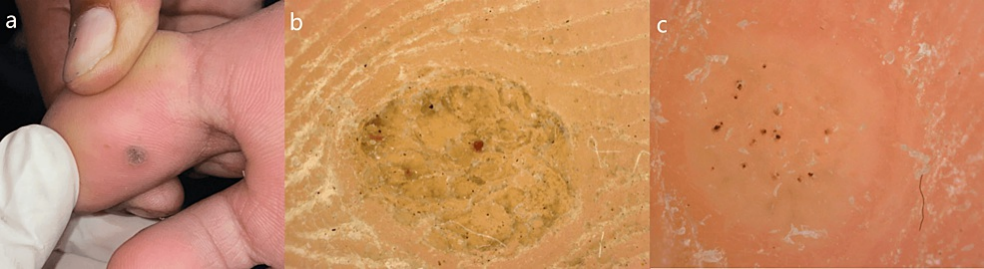
The images of plantar wart. The images showing (from left to right) (a) clinical image of plantar wart, (b) non-contact polarized dermoscopic image of unpared lesion showing red dots and interrupted dermatoglyphics, and (c) non-contact polarized dermoscopic image of pared lesion showing red dots and interrupted dermatoglyphics (10×).

A total of 70 patients were recruited in our study. Mean age of patients with callus, corn, and wart was 42±11.85 years, 32.29±11.15 years, and 32.42±13.51 years, respectively. Overall, majority of patients were male (52.9%) pertaining to age group interval of 19-30 years (42.9%). Callus and corn cases were males in majority (32.4% and 21.6%, respectively), while warts cases had females in majority (57.6%). A majority of patients had warts (51.4%) and the rest were suffering from calluses (28.6%) and corns (20%). All patients of callus as well as corn had absent homogenous black/red dots in unpared and pared lesions, while they were present in 100% unpared and pared cases of warts (Figures 1a-1c).

**Figure 2:**
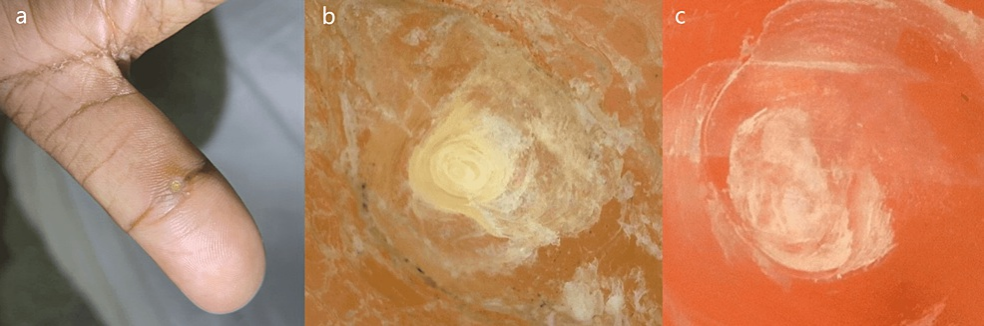
The images of palmar corn. The images showing (from left to right) (a) clinical image of palmar corn, (b) non-contact polarized dermoscopic image of unpared lesion showing central keratin white area, and (c) non-contact polarized dermoscopic image of pared lesion showing translucent central core (10×).

In all patients with calluses and corns, papilliform surfaces were absent in both unpared and pared lesions, while in 55.56% of unpared and 66.67% of pared cases of warts, papilliform surfaces were present. All patients of callus had intact skin lines in unpared and pared lesions. Skin lines were interrupted in 14.29% unpared and pared cases of corn and all lesions of warts. Majority of patients had absent red linear vessels in 100% unpared and pared cases of callus as well as corn, while presence in 16.67% unpared and 30.56% pared cases of warts. All patients with callus and wart had absent translucent central core in unpared and pared lesions, whereas it was present in 92.85% unpared and 100% pared lesions of corn (Figures 2a-2c).

**Figure 3:**
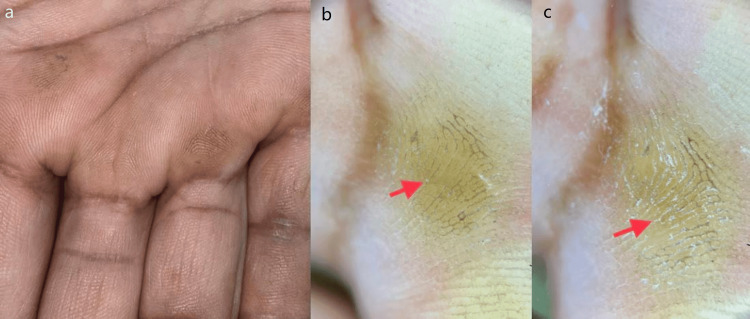
The images of palmar callus. The images showing (from left to right) (a) clinical image of palmar callus, (b) non-contact polarized dermoscopic image of unpared lesion showing homogenous opacity with intact dermatoglyphics, and (c) non-contact polarized dermoscopic image of pared lesion showing homogenous opacity with intact dermatoglyphics (10×).

The majority of patients had homogenous opacity in 75% unpared and 100% pared cases of callus whereas it was absent in all unpared and pared lesions of corn as well as wart (Table 1; Figures 3a-3c). No association between unpared and pared lesions was found (p>0.05).

**Table 1:** Dermoscopic features in unpared and pared lesions of warts, corns, and calluses.

Variables	Wart, n=36		Corn, n=14		Callus, n=20	
	Unpared	Pared	Unpared	Pared	Unpared	Pared
Homogenous black/red dots	36 (100%)	36 (100%)	0	0	0	0
Papilliform surface	20 (55.56%)	24 (66.67%)	0	0	0	0
Interrupted skin lines	36 (100%)	36 (100%)	2 (14.29%)	2 (14.29%)	0	0
Red linear vessels	6 (16.67%)	11 (30.56%)	0	0	0	0
Translucent central core	0	0	1 (7.15%)	14 (100%)	0	0
Homogenous opacity	0	0	0	0	15 (75%)	20 (100%)

## Discussion

In dermatology practice, palmoplantar warts, corns, and calluses are frequently seen, which can make it difficult to carry out daily tasks. The three most typical plantar lesions are corns, calluses, and plantar warts [4]. Although they can typically be diagnosed clinically, there may be some doubt in some circumstances [5]. With the naked eye, it can occasionally be challenging to distinguish a plantar wart from a callus or corn. Differentiating them is likewise a difficult task [6]. In the past 10 years, dermoscopy has been more frequently used to assist in the identification of a variety of dermatological problems. It is being used more often to help with the diagnosis of different infectious dermatoses [7]. The structures that comprise the epidermis and dermal papilla can be seen during dermoscopy which makes the skin's top layer, the stratum corneum translucent. Dermoscopy can be utilized as an auxiliary tool for the confirmation of the diagnosis in doubtful instances, particularly when the patient refuses or is unable to undergo a histopathological investigation [8]. It can be used without running the danger of spreading infection, which is crucial when treating infections like warts [9]. There are few literature-based dermoscopic descriptions of the aforementioned conditions [10]. Here, we attempted to appraise the dermoscopic findings in pared and unpared cases of palmoplantar warts, corns, and calluses.

In the present study, cases from age group interval of 19-30 years had maximum cases (42.9%). The mean age of patients diagnosed with callus, corn, and wart was 42±11.85 years, 32.29±11.15 years, and 32.42±13.51 years, respectively. Gender-wise evaluation appraised slight male sex predilection with majority of males (52.9%) and rest females (47.1%). Age and sex can both be thought of as proxies for unmeasured underlying causative factors, such as variations in men's and women's footwear and rising age-related prevalence of typical co-morbidities. In the study conducted by Al Rudaisat and Cheng, patients' average ages were 36±15.3 years. The mean age of instances of common warts was 51.3 years, and the mean ages of cases of palmar and plantar warts were 34.1±11.3 years and 29.5±5.7 years, respectively [11]. Aqil et al. also appraised the average age of 32.8 years (10-65) for the cases of warts [12]. These results were in consensus with our study data and strengthened our results.

Dermoscopic characteristics of common warts were characterized by Lallas et al. as tightly clustered papillae with a central red dot or loop encircled by whitish haloes [13]. Dermoscopy of palmoplantar warts frequently shows numerous obvious hemorrhages inside a well-defined, yellowish papilliform surface where skin lines are broken, according to published reports. This pattern allows them to be distinguished from callus, which lacks blood spots and instead displays a center reddish to blue structure less pigmentation [14]. Furthermore, four distinct dermoscopic patterns-unspecific, fingerlike, mosaic, and knoblike patterns which can occur in a single wart were discovered in a study involving a significant number of individuals [15].

Corn had a yellow area, a whitish annular band, and a translucent inner center, according to dermoscopy by Ankad et al. It was typical to find dermatoglyphics preserved. The yellow or white halo and red dots were noticeably lacking. Under dermoscopy, the stratum corneum thickening manifests as a yellow region. Localized fibrosis is the cause of the central transparent core that serves as the nucleus of the corn. The white ring and prominent collagen thickening are interrelated which similar to the findings of previous reports [1,4]. The callus was also clarified by Ankad et al. under dermoscopy, which revealed a noticeable opaque yellowish region with retention of dermatoglyphics. There were observed focal white regions [1]. These results are consistent with Sonthalia et al's earlier description [16]. In contrast to plantar warts, there were no vascular components. Hyperkeratosis, orthokeratosis, and acanthosis caused the yellow spots. Focal white spots that resembled white scales were caused by focal parakeratosis.

Plantar warts had vascular components, although corn and callus did not. It results from dermal papillae that extend upward and have dilated and thrombosed capillaries [1]. Even though plantar papule paring may show thrombosed vessels, the non-invasive evaluation of vessels for the distinction among different lesions and the confirmation of clinical diagnoses using a dermoscope is very useful. The therapeutic prognosis of a plantar wart is significantly influenced by these vascular patterns. The resolution of the lesion is shown by the removal of dotted vessels [9].

## Conclusions

The accuracy of identifying various clinical forms of cutaneous warts, as well as assistance in separating them from other similar skin lesions like calluses and corns may be enhanced by dermoscopy without paring. It can be a quick clinical diagnostic tool in distinguishing it from close differentials. Therefore, dermoscopy, as a non-invasive diagnostic aid, can annihilate the anxiety related to invasive procedures like skin biopsy. Although it is a prefatory and introductory study, we recommend more quantitative research to substantiate and corroborate the benefits of dermoscopy.
